# The Effect of Foot Deformity and First Metatarsophalangeal Joint Plantar Pain on Performance in DanceSport Athletes

**DOI:** 10.3390/children9081169

**Published:** 2022-08-04

**Authors:** Zijian Liu, Hiroki Yabiku, Takumi Okunuki, Shuo Chen, Takuma Hoshiba, Toshihiro Maemichi, Hideaki Nagamoto, Yanshu Li, Tsukasa Kumai

**Affiliations:** 1 Graduate School of Sport Sciences, Waseda University, Saitama 359-1192, Japan; 2 Waseda Institute for Sport Sciences, Waseda University, Saitama 359-1192, Japan; 3 Faculty of Sport Sciences, Waseda University, Saitama 359-1192, Japan; 4 Graduate School of Human Sciences, Waseda University, Saitama 359-1192, Japan

**Keywords:** adolescent DanceSport athlete, hallux valgus angle, 1st MTP joint plantar pain, plantar pressure distribution

## Abstract

Background: Adolescent DanceSport athletes who regularly dance in high heels have a higher possibility of developing hallux valgus deformity and foot pain. We believe that the occurrence of foot disorders may change the loading on their feet, which thus affects the athletic performance of those adolescents. Methods: A total of 63 adolescent DanceSport athletes (16 boys, 47 girls) were included. The plantar pain in the first metatarsophalangeal (1st MTP) joint was evaluated using a questionnaire, and the hallux valgus angle was evaluated using digital photographs (HVAp). The loading values of the plantar pressure while performing relève on demi-pointe were measured using sensor sheets. The participating boys and girls were analyzed separately. Results: The results showed that female adolescent DanceSport athletes with the 1st MTP joint plantar pain showed a decrease in the loading distribution and plantar pressure percentage on the hallux and an increased loading distribution and pressure distribution of the metatarsal head as the HVAp increased. Conclusion: Among adolescent DanceSport athletes with plantar pain in the 1st MTP joint and a large HVAp, the loading manner of the foot may have changed, which may be associated with a decrease in the toe function and performance.

## 1. Introduction

Hallux valgus deformity occurs in the first metatarsophalangeal (1st MTP) joint, with higher prevalence in women. Hallux valgus deformity has been associated with aging and high-heeled shoes [1,2]. In particular, foot disorders have been frequently reported among dancers and have received increased clinical attention recently [3,4]. Specifically, hallux valgus deformity was prevalent among ballet dancers and has been suggested to occur when the load on the 1st MTP joint increases incorrectly due to an increased medial pressure on the hallux by inward turning of the foot during external rotation of the lower extremity [5,6]. The plantar pressure is laterally deviated in the gait of a non-athletic individual, depending on whether hallux valgus deformity is present [7]. Among dancers, hallux valgus deformity has been shown to affect knee–foot alignment and dance performance [8]. On the other hand, as the heel height increases, the forefoot experiences increased pressure, especially on the first metatarsal head [9]. Such instances of high pressure on the forefoot coupled with increased plantar pressure can lead to pain at the MTP joints [10,11].

Adolescent athletes who aspire to become professional DanceSport performers dance in high-heeled shoes, which are associated with plantar pain in the 1st MTP joint and the development of hallux valgus deformity. In particular, a survey of the incidence of subjective foot symptoms with DanceSport athletes showed a high prevalence of disorders of the lower limbs or foot, especially joint disorders [12]. According to the previous studies, foot symptoms or hallux valgus deformity can influence the dancer’s performance. However, there are few studies on the plantar pain of the 1st MTP joint and the hallux valgus in dancers, and the changes in the loading distribution and plantar pressure percentage was unclarified based on the area of the foot that was divided.

Relevé on demi-pointe is a basic movement in dance [13,14] that requires a dancer’s balance, intrinsic receptivity, and other performance characteristics [15], regardless of the dance discipline. Since this movement involves the functionality of the feet, we deemed it useful for assessing the toe function and performance among dancers.

This study aimed to clarify the effect of plantar pain in the 1st MTP joint and the hallux valgus on the loading distribution and plantar pressure percentage while a dancer performed relevé on demi-pointe.

## 2. Materials and Methods

### 2.1. Participants Characteristics

**Figure 1:**
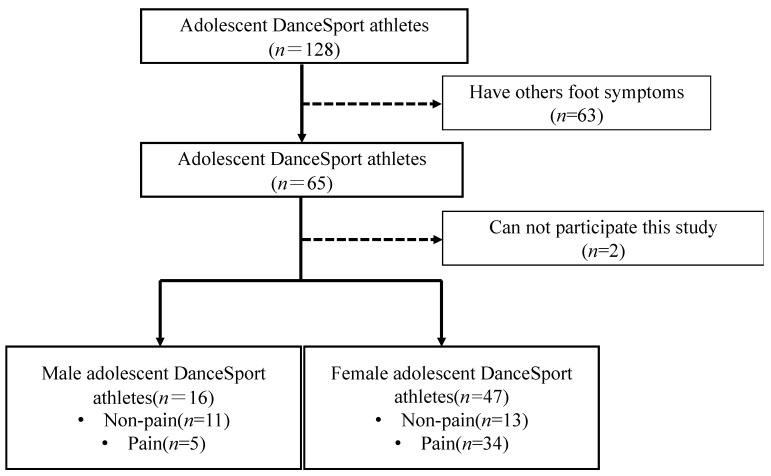
Process flowchart for the classification of groups based on the participants’ conditions.

In this study, we examined 63 adolescent DanceSport athletes from a DanceSport school in China who participated in national and international competitions. The survey questionnaire included questions regarding basic and demographic information age (male: 16.0 ± 1.4 years, Female: 15.8 ± 1.4 years), weight (male: 56.0 ± 8.0 kg, Female: 48.7 ± 4.8 kg), athletic career (male: 3.3 ± 2.0 years, Female: 2.7 ± 1.6 years), daily practice time(male: 4.9 ± 1.0 h, Female: 5.0 ± 1.1 h) and assessed whether plantar pain was present in the 1st MTP joint on the right foot when walking, practicing or competing. To make sure the athletes who only had 1st MTP plantar pain were included in this study, the survey asked questions regarding other symptoms. Based on the results, we excluded DanceSport athletes who had foot symptoms other than 1st MTP plantar pain from 128 adolescent DanceSport athletes (Figure 1).

The parent/guardian provided written informed consent for the study on the adolescent participants after the purpose of the study, research methods, and ethical considerations were explained to them. This study was approved by the Ethical Review Committee on Research Involving Human Subjects of Waseda University.

### 2.2. Survey of Plantar Pain in the 1st MTP Joint and Hallux Valgus Measurement

After classifying the participants based on sex, DanceSport athletes with plantar pain in the 1st MTP joint were classified as the pain group, while those without that pain were classified as the non-pain group. We examined whether there were differences in the loading and plantar pressure distribution by grouping them according to the presence of plantar pain in the 1st MTP joint, and analyzed the relationship between HVAp and loading distribution and plantar pressure percentage. In addition, we analyzed the relationship between the HVAp in pain group with the loading distribution and plantar pressure percentage.

For measurement of hallux valgus angle, we used a method based on foot photographs (HVAp) [16]. The participants spread both feet shoulder-width apart and loaded both feet as equally as possibly. They stood up straight with their hands on their anterior superior iliac spine and their eyes looking forward. A measurement camera (Canon, SX430is) was set at a 15° inclined angle relative to a vertical line passing through the tip of the second toe, and a still screen was obtained.

**Figure 2:**
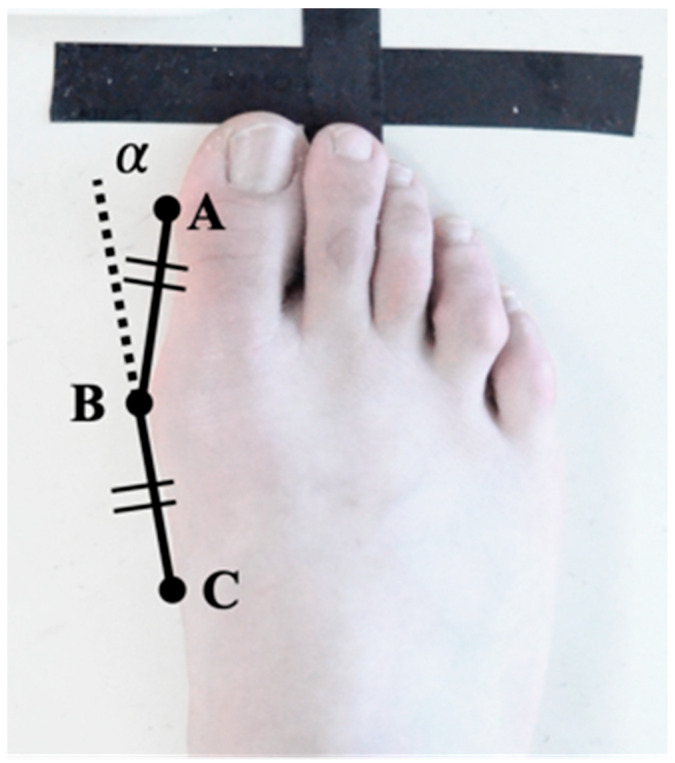
Measurement method for the hallux valgus angle.

As for HVAp measurement, we used the ImageJ software (Bethesda, MA, USA). First, the tangent line (AB) was drawn from the medial edge of the big toe (A) to the inside edge of the head of the first metatarsal bone (B). About point C, where lines AB and BC are the same distance from the contact point (B) to the along the medial side of the first metatarsal. The angle α between lines AB and BC was the metatarsal angle [16] (Figure 2).

### 2.3. Plantar Pressure Measurement 

#### Equipment

**Figure 3:**
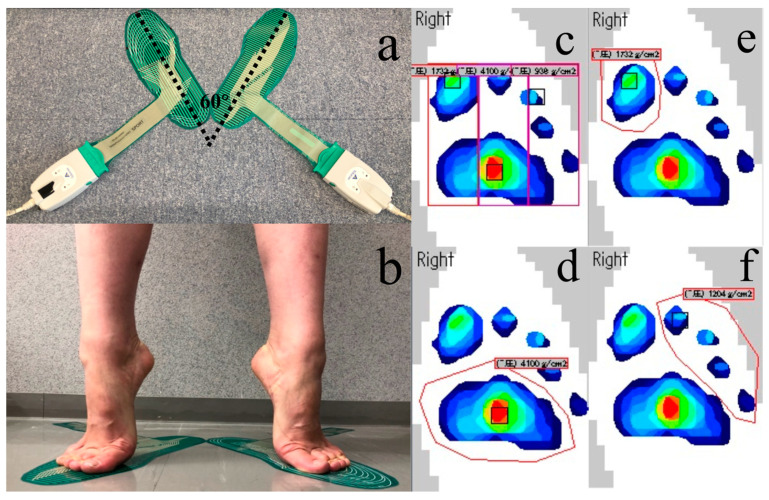
Experimental set-up showing (**a**) the installation of the sensor; (**b**) the limb positions (relevé on demi-pointe); (**c**) medial, middle, lateral from lift; (**d**) metatarsals hand; (**e**) hallux area and (**f**) toe area.

A film-type pressure distribution measurement system (F-scan II; Tekscan, Japan) was used to measure the distribution of plantar pressure. A line at the position where the front-to-back width of the sensor sheets (two sheets) was maximal was set as the foot reference line. Following the DanceSport habits, we set the foot reference line on flat sensor at 30° external rotation to the sagittal plane (60° in total) of the participant (Figure 3a).

The participant stood with the second toe and mid-heel aligned with the foot reference line on the sheet, and then slowly moved into the relevé on demi-pointe position, holding the position for 10 s (500 frames).

Limb positions: to reproduce plantar pressure in the plantar flexion position during dancing, plantar pressure was evaluated using the relevé on demi-pointe (Figure 3b), with the knee joint in extension and both hands on the hips. Two patterns were used to divide the area for the data analysis:

A. Trisection

The medial and lateral widths of the tangents to the toes were trisected into three areas: medial, middle, and lateral from the left, as shown in Figure 3c.

B. By site

The ground contact surface was divided into three areas according to the parts of the foot: metatarsals hand (Figure 3d), hallux (Figure 3e) and toe (Figure 3f).

C. Data processing

Of the 500 frames that were measured for 10 s, 300 frames with stable values were extracted. The pressure data were extracted from measurements performed at each sensing point on the sensor sheet, and an image of one frame was created by averaging the pressure data of 300 frames for each sensing point. This one-frame image was divided into several regions using the below method, and the loading distribution (%mass) was calculated as a ratio of the load applied to each area to the total load applied to the right foot.

Likewise, the plantar pressure percentage was calculated for each area (%pressure impulse) as a ratio between the pressure applied on each area to the total pressure applied on the right foot.

### 2.4. Statistical Analysis

For variables with a normal distribution, the independent sample parametric test was used to compare the means of two groups (pain vs. non-pain) in male or female adolescent DanceSport athletes. For non-parametric test data, the Mann–Whitney test was used to compare data between pain and non-pain in male or female participants.

Pearson’s correlation coefficient was used to determine the relationship between the HVAp and the loading distribution and the plantar pressure percentage in each area in the pain and non-pain groups. 

About HVAp measurement, in first to estimate intra-reliability of hallux valgus angle measurement with graph, we used intraclass correlation coefficient (ICC1,3) estimates. Reliability thresholds for ICC values were defined as poor (<0.50), moderate (0.50–0.75), good (0.75–0.90) and excellent (>0.90). In this study, ICC (1,3) values was 0.941.

The statistical software SPSS Statistics Ver.24 (SPSS Inc.; Chicago, IL, USA) was used for statistical processing, and the significance level was set at *p* < 0.05.

## 3. Results

**Figure 4:**
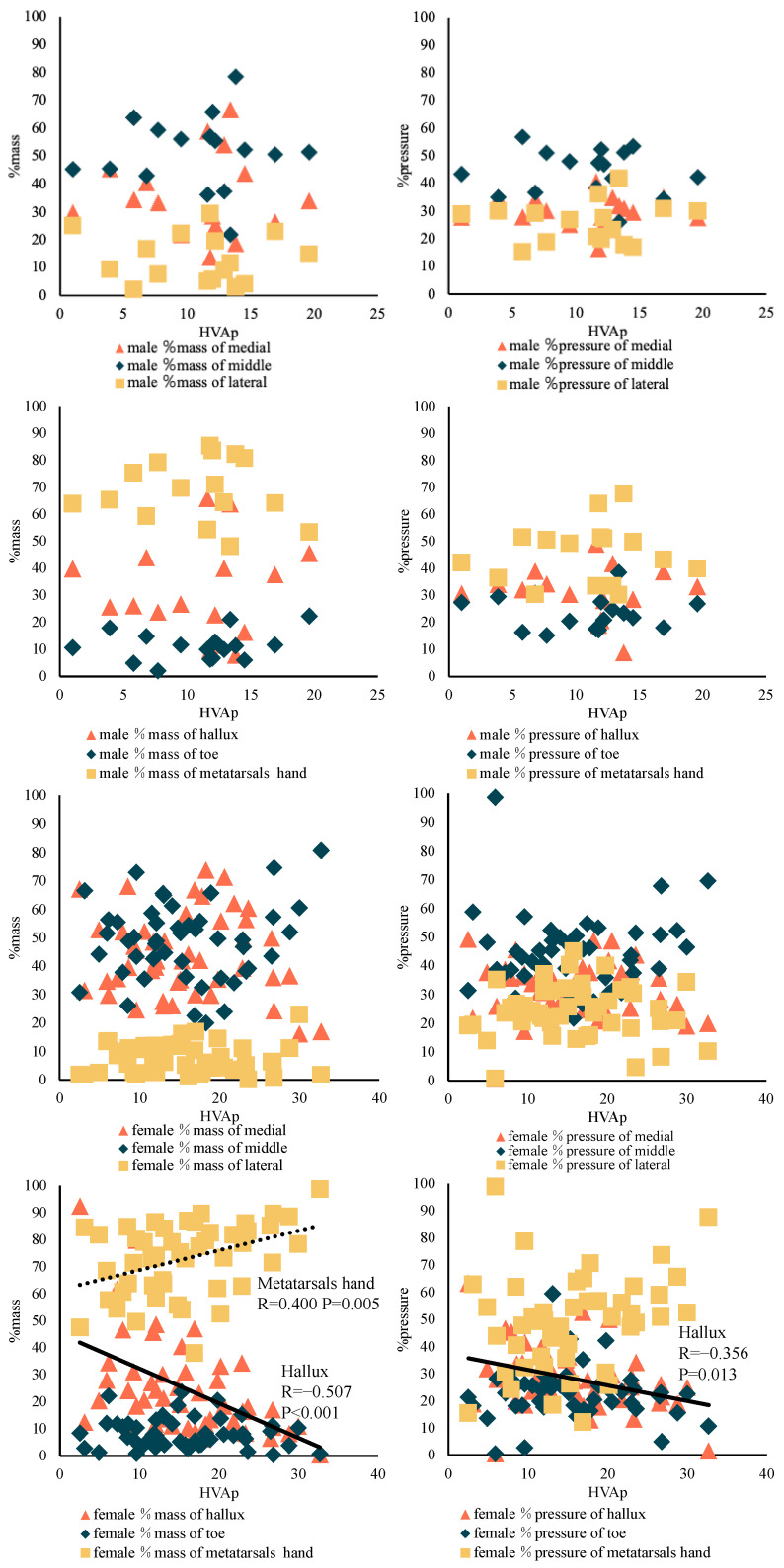
Relationship between the hallux angle and the %mass and %pressure impulse in male and female DanceSport athletes.

In adolescent DanceSport athletes, regardless of sex, there was no significant difference in the %mass and %pressure impulse in the medial, middle, lateral, hallux, toe and metatarsals between the pain and non-pain groups. Among male adolescent DanceSport athletes, there was no significant relationship between increasing HVAp and the %mass and %pressure impulse in the medial, middle, lateral, hallux, toe and metatarsals. Among female adolescent DanceSport athletes, the increased HVAp significantly decreased the %mass in the hallux (*p* < 0.001, R = −0.507) as well as the %pressure impulse (*p* = 0.013, R = −0.356) and significantly increased the %mass in the metatarsals hand (*p* = 0.005, R = 0.400) (Figure 4).

**Figure 5:**
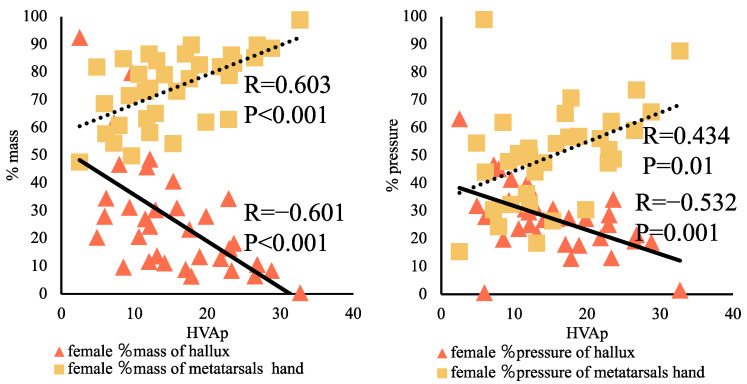
Relationship between the hallux angle and the %mass and %pressure impulse in females with first metatarsophalangeal joint plantar pain.

The relationship between the %mass and %pressure impulse in the metatarsal and hallux in female adolescent DanceSport athletes with or without 1st MTP joint plantar pain was examined. In the hallux, the %mass (*p* < 0.001, R = −0.601) and %pressure impulse (*p* = 0.001, R = −0.532) in the pain group were significantly correlated; it was also significantly correlated with the %mass (*p* < 0.001, R = 0.603) and the %pressure impulse (*p* = 0.01, R = 0.434) in the metatarsals hand (Figure 5). 

## 4. Discussion

The results of this study showed that the HVAp or 1st MTP joint plantar pain had no significant relationship with either the lateral, middle or the medial loading distribution of the forefoot or the plantar pressure percentage in adolescent DanceSport athletes of either sex. Although the relationship between foot pain and plantar pressure has been well examined, the relationship between plantar pain of the 1st MTP joint and plantar pressure remains scarcely investigated. Other studies have reported that foot pain may lead to the avoidance of the painful area during running [17]. However, the results of this study showed no significant relationship between lateral, middle and medial load distributions and plantar pressure among adolescent DanceSport athletes with the 1st MTP joint plantar pain in males and females, suggesting that pain was probably not avoided. The participants in this study were high-level adolescent DanceSport athletes and, therefore, the presence or absence of the 1st MTP joint plantar pain did not change their standing position in the demi-pointe for the performance.

Galica et al. [18] reported that the maximum force and peak pressure on the lateral side of the metatarsus decreased significantly when a hallux valgus deformity occurred. Another study has shown that in normal individuals with painless hallux valgus, the lateral plantar pressure during running decreases, and the loading in the medial foot and hallux increases as the degree of hallux valgus increases [19]. In this study, only female adolescent DanceSport athletes showed that with an increase in HVAp, the metatarsal underwent changes in the loading distribution, leading to the decrease in plantar pressure to the hallux. Female adolescence DanceSport athletes seem to be more susceptible to the effects of hallux valgus.

Furthermore, in this study, the correlation between the HVAp and the loading distribution and plantar pressure in the hallux and metatarsal were higher in the pain group. As shown in a previous study, the pressure on the hallux may decrease as the hallux valgus angle increases during running, and it is even lower in painful hallux valgus than in painless hallux valgus [20]. Particularly in dancers, hallux valgus, sesamoid disorder and first metatarsal head fractures are more prevalent and are associated with pain around the first MTP joint [3,21,22]. Moreover, hallux valgus occurs as a disorder in the 1st MTP joint, and the muscles (abductor hallucis and flexor hallucis brevis) may be separated from its original position while simultaneously experiencing dysfunction [2,4,23]. Our results revealed that in female adolescent DanceSport athletes in the pain group, the loading distribution and plantar pressure on the hallux further decreased and the loading value and pressure on the metatarsal increased as the HVAp increased. Mira et al. [24] reported that the function of the hallux played an important role for ballet dancers, and our results suggest that in female adolescent DanceSport athletes with plantar pain in the 1st MTP joint and increasing HVAp, the function of the 1st MTP joint may have been affected.

There were limitations to this study. We required all participants to perform relevé on demi-pointe to their best ability. However, we did not control the ankle flexion angle. The deviation in the angle of ankle flexion may have caused loading distribution changes. For dancers performing relevé on demi-pointe, the ankle movement comprises more movements than just flexion, and changes in the ankle movement must be measured. On the other hand, DanceSport athletes wear high heels during dancing. However, in this study, we used the relevé on demi-pointe to examine performance with adolescent DanceSport athletes. In the future, it will be necessary to investigate how function and performance are affected by foot disorders and hallux valgus with female DanceSport athletes during adolescence using high heels. Conversely, we did not explain the correlation between HVAp and foot plantar pressure in male DanceSport athletes, and we could not find any previous study where male dancers’ performance were discussed, particularly regarding their foot plantar pressure distribution. In the future, we should aim to clarify why male DanceSport athletes are not affected by hallux valgus and 1st MTP joint plantar pain.

## 5. Conclusions

The loading distribution and plantar pressure did not change when plantar pain occurred in the 1st MTP joint for adolescent DanceSport athletes. Among adolescent DanceSport female athletes, the loading distribution and plantar pressure were displaced from the hallux to the metatarsal as the HVAp increased; this effect was increased if a female dancer had plantar pain in the 1st MTP joint.

We had expected an effect on the performance with a change in the foot landing. We conclude that dance motions including the foot injury condition of female adolescent DanceSport athletes need to be analyzed in the future.

## Data Availability

Not applicable.

## References

[1] Piqué-Vidal C., Solé M.T., Antich J. (2007). Hallux Valgus Inheritance: Pedigree Research in 350 Patients With Bunion Deformity. J. Foot Ankle Surg..

[2] Hecht P.J., Lin T.J. (2014). Hallux Valgus. Med. Clin. N. Am..

[3] Kennedy J.G., Collumbier J.A. (2008). Bunions in Dancers. Clin. Sports Med..

[4] Gamboa J.M., Roberts L.A., Maring J., Fergus A. (2008). Injury Patterns in Elite Preprofessional Ballet Dancers and the Utility of Screening Programs to Identify Risk Characteristics. J. Orthop. Sports Phys. Ther..

[5] Prochazkova M., Tepla L., Svoboda Z., Janura M., Cieslarová M. (2014). Analysis of foot load during ballet dancers’ gait. Acta Bioeng. Biomech..

[6] Biz C., Favero L., Stecco C., Aldegheri R. (2012). Hypermobility of the first ray in ballet dancer. Muscle Ligaments Tendons J..

[7] Wen J., Ding Q., Yu Z., Sun W., Wang Q., Wei K. (2012). Adaptive changes of foot pressure in hallux valgus patients. Gait Posture.

[8] Seki H., Miura A., Sato N., Yuda J., Shimauchi T. (2020). Correlation between degree of hallux valgus and kinematics in classical ballet: A pilot study. PLoS ONE.

[9] Ko P.-H., Hsiao T.-Y., Kang J.-H., Wang T.-G., Shau Y.-W., Wang C.-L. (2009). Relationship Between Plantar Pressure and Soft Tissue Strain under Metatarsal Heads with Different Heel Heights. Foot Ankle Int..

[10] Besse J.-L. (2017). Metatarsalgia. Orthop. Traumatol. Surg. Res..

[11] Hashmi F., Nester C.J., Wright C.R., Lam S. (2016). The evaluation of three treatments for plantar callus: A three-armed randomised, comparative trial using biophysical outcome measures. Trials.

[12] Pellicciari L., Piscitelli D., De Vita M., D’Ingianna L., Bacciu S., Perno G., Lunetta L., Rosulescu E., Cerri C.G., Foti C. (2016). Injuries Among Italian DanceSport Athletes: A Questionnaire Survey. Med. Probl. Perform. Artist..

[13] Moser B.R. (2011). Posterior Ankle Impingement in the Dancer. Curr. Sports Med. Rep..

[14] Russell J.A., Shave R.M., Kruse D.W., Nevill A.M., Koutedakis Y., Wyon M.A. (2011). Is Goniometry Suitable for Measuring Ankle Range of Motion in Female Ballet Dancers? An Initial Comparison With Radiographic Measurement. Foot Ankle Spec..

[15] Shah S. (2009). Determining a Young Dancer′s Readiness for Dancing on Pointe. Curr. Sports Med. Rep..

[16] Omae H., Ohsawa T., Hio N., Tsunoda K., Omodaka T., Hashimoto S., Ueno A., Tajika T., Iizuka Y., Chikuda H. (2021). Hallux valgus deformity and postural sway: A cross-sectional study. BMC Musculoskelet. Disord..

[17] Keijsers N., Stolwijk N., Louwerens J., Duysens J. (2013). Classification of forefoot pain based on plantar pressure measurements. Clin. Biomech..

[18] Galica A.M., Hagedorn T.J., Dufour A.B., Riskowski J.L., Hillstrom H.J., Casey V.A., Hannan M.T. (2013). Hallux valgus and plantar pressure loading: The Framingham foot study. J. Foot Ankle Res..

[19] Martínez-Nova A., Rodríguez R.S., Pérez-Soriano P., Llana-Belloch S., Leal-Muro A., Pedrera-Zamorano J.D. (2010). Plantar pressures determinants in mild Hallux Valgus. Gait Posture.

[20] Nishimura A., Ito N., Nakazora S., Kato K., Ogura T., Sudo A. (2018). Does hallux valgus impair physical function?. BMC Musculoskelet. Disord..

[21] Einarsdottir H., Troell S., Wykman A. (1995). Hallux Valgus in Ballet Dancers: A Myth?. Foot Ankle Int..

[22] Kadel N. (2014). Foot and Ankle Problems in Dancers. Phys. Med. Rehabil. Clin. N. Am..

[23] Perera A., Mason L., Stephens M. (2011). The Pathogenesis of Hallux Valgus. J. Bone Jt. Surg..

[24] Mira N.O., Marulanda A.F.H., Peña A.C.G., Torres D.C., Orrego J.C. (2019). Study of Ballet Dancers During Cou-De-Pied Derrière with Demi-Plié to Piqué Arabesque. J. Dance Med. Sci..

